# Micronutrients in infants suffering from cow’s milk allergy fed with dietary formulas and breast milk

**DOI:** 10.1186/s12887-024-04591-8

**Published:** 2024-02-13

**Authors:** Shohreh Maleknejad, Kobra Dashti, Afshin Safaei-Asl, Zahra Atrkar Roshan, Soodeh Salehi, Afagh Hassanzadeh-Rad

**Affiliations:** Pediatric Diseases Research Center, Guilan University of Medical Sciences, Rasht, Iran

**Keywords:** Cow’s milk allergy, Micronutrients, Breastfeeding, Formula

## Abstract

**Introduction:**

Cow’s milk allergy (CMA) is the most common food allergy in infants. As this food allergy indicates a wide range of clinical syndromes due to immunological reactions to cow’s milk proteins, we aimed to evaluate the status of micronutrients in infants suffering from cow’s milk allergy.

**Methods:**

In this historical cohort study, infants with CMA were divided into two equal groups: breastfeeding and diet formula feeding. Data were gathered by a form, including the micronutrients such as iron, selenium, calcium, phosphorus, zinc, and vitamin D. Groups were compared and data were analyzed by the IBM SPSS version 21.

**Results:**

This study involved 60 six-month-old infants, and the findings revealed no significant difference between the two groups concerning magnesium, phosphorus, zinc, and vitamin D. However, infants in the formula-feeding group exhibited significantly elevated mean serum levels of iron and selenium, whereas breastfed infants displayed higher levels of calcium.

**Conclusion:**

The findings of this research revealed a significant difference in calcium, selenium, and iron levels between formula-fed and breastfed infants, even though all variables were within the normal range for both groups. In light of these results, conducting further studies with a larger sample size and extended follow-up periods becomes imperative.

## Introduction

The prevalence of allergic reactions to foods in the first three years of life is about 6–8% [1]. Also, it has been noted that allergy to cow’s milk is the most common form of food allergy in children. Dairy products consumed by mothers can also cause allergies in infants [2]. Food allergies are often caused by IgE-mediated and non-IgE-mediated mechanisms [3] that may lead to growth disorder, anemia, colic, eczema, chronic diarrhea, diarrhea, hemorrhage, persistent vomiting, constipation, rhinorrhea, wheezing, etc. [4]. .

Cow’s milk has several protein compounds that can enter the breast milk and induce digestive problems [5]. On the other hand, Breast milk is the most appropriate source of food which ensures the physical and brain development of a child up to the age of 6 months [6] and reduces malnutrition and deaths in children and infants [7, 8]. Therefore, in patients with food allergies, it is primarily recommended to continue breastfeeding by adjusting the mother’s diet. However, in irresponsive infants or mothers with the inability to breastfeed, an appropriate formula is needed. In these cases, hypoallergenic formulas such as extensively hydrolyzed formula (eHF) or amino acid-based formulas can be the choice [9].

Notably, infants have a constant need for micronutrients and sufficient nutrition for growth and development. Since cessation of consuming dairy products in breastfeeding mothers with allergic infants is a necessary treatment, one of the most important concerns is the lack of receiving these micronutrients from mother’s milk. Considering the high prevalence of food allergy and the importance of proper nutrition in this age group, paying attention to micronutrients is an important and challenging issue. Therefore, it may be assumed that the use of formulas containing micronutrients is more suitable for these infants. As it seems that breast milk is the appropriate milk for any type of breastfeeding and based on the shortage of evidence and similar studies comparing micronutrients in breast and formula-fed infants, we assessed the status of micronutrients in infants with cow’s milk allergy (CMA) in these infants.

## Materials and methods

This historical cohort study was conducted from October 2020 to October 2021 on 60 infants diagnosed with CMA who had been referred to the gastroenterology clinic of 17 Shahrivar Hospital, Iran. CMA was indicated with the clinical symptoms and signs of sensitivity to cow protein such as vomiting, chronic diarrhea, dysentery, and restlessness. A definitive diagnosis of CMA was indicated by the symptom elimination due to the cessation of cow’s milk from the mother’s and infant’s diet and the recurrence of symptoms upon reintroduction of milk [10]. Infants who received this diet for at least 3 months entered the study.

Exclusion criteria were underlying diseases or a history of taking drugs or supplements other than those mentioned as necessary for age. Also, infants with combined feeding including breast milk and dietary formula, those who started complementary feeding earlier than 6 months of age, and breastfed infants whose mother had a special medical condition or needed to take a special drug or supplement except Calcium- vitamin D were excluded. We also did not include infants with failure to thrive based on the WHO growth standards with z-scores less than two standard deviations.

Notably, the age range of our infants was less than 6 months and we selected those with gastrointestinal manifestations, including food and protein-induced enterocolitis, proctocolitis, and enteropathy syndromes.

This study comprised 60 infants, with 30 infants assigned to the breastfeeding group and 30 to the formula-feeding group. In the breastfeeding group (group 1), mothers adhered to a cow’s milk-restricted diet and received a daily supplement of Calcium-vitamin D. For the formula-feeding group (group 2), infants exclusively consumed extensively hydrolyzed milk powder, excluding those on amino acid-based milk powders. Both groups received daily supplements of 400 units of vitamin D and 1500 units of vitamin A.

At 6 months of age, before the introduction of complementary feeding, all infants underwent laboratory assessments. These included plasma levels of calcium, phosphorus, iron, vitamin D, magnesium, zinc, and selenium in a unified laboratory setting. Plasma levels of magnesium, zinc, iron, and calcium were determined using the Audit unit kit, Ferene, Colorimetric, Xylidyl Blue, and Arsenazo methods, respectively. The level of 25-hydroxyvitamin D was assessed with the Diasorine kit utilizing the Chemiluminescence method. Serum phosphorus levels were measured using the Phosphomolibdate method with a Man kit, and selenium plasma levels were determined through the Atomic Absorption method.

### Statistical analysis

Data were analyzed by IBM SPSS Statistics for Windows, Version 20.0. Armonk, NY: IBM Corp. At first, the normality of the quantitative data was assessed using the Kolmogorov-Smirnov test. In the case of normally- distributed data, T-test was used to compare the variables in two groups. If there were non-normally distributed variables, the Mann–Whitney U test was used. A significance level of less than 0.05 was considered significant.

## Results

In this study, 60 infants under 6 months known to have CMA were evaluated in two groups of dietary formula-fed and breast milk. Results showed that 21 neonates (70%) of dietary formula-fed and 16 (53.3%) of the breastfed group were girls and no significant difference was noted between the two groups in terms of sex (*P* = 0.288). In terms of weight at six months, there was no significant difference between dietary formula (7583 ± 3.920 g) and breast milk (7746 ± 6.859 g) (*P* = 0.48).

As Table 1 shows, it was found that the mean level of serum calcium in breast-fed infants was significantly higher than the serum calcium level of infants fed with dietary formula (*P* = 0.036). Meanwhile, the mean serum level of selenium and iron in formula-fed infants was significantly higher than in the breastfed group (*P* = 0.0001). Notably, our results indicated that despite these significant differences between calcium, selenium, and iron, all of them were within the normal range in both groups. However, in terms of phosphorus, zinc, and vitamin D, the comparison of the two groups showed no significant difference (*P* > 0.05).

**Table 1:** Comparison of the mean serum levels of micronutrients in the two groups

Micronutrients	Formula *N* = 30	Breastfeeding *N* = 30	*P*-value
Calcium ( mg/dL)	9.94 ± 0.37	10.17 ± 0.47	0.036
Magnesium ( mg/dL)	2.34 ± 0.18	2.33 ± 0.16	0.712
Phosphorus ( mg/dL)	5.65 ± 0.72	5.60 ± 0.62	0.775
selenium ( mg/dL)	87.42 ± 25.63	64.65 ± 19.08	0.0001
zinc ( mg/dL)	93.07 ± 18.37	92.87 ± 16.15	0.964
Iron ( mg/dL)	70.70 ± 26.02	43.27 ± 20.69	0.001
Vitamin D ( mg/dL)	34.85 ± 10.97	39.00 ± 17.31	0.273

## Discussion

Cow’s milk is one of the first foods that enter a child’s diet, and CMA is the most common food allergy in the first year of life [11]. Conducted due to its high prevalence and the widespread necessity of identifying optimal nutrition for infants, this study yielded insightful results. Despite both groups exhibiting all variables in the normal range, breastfed infants displayed a significantly higher mean calcium level compared to formula-fed infants. While no significant differences were observed between groups in terms of phosphorus, zinc, and vitamin D, formula-fed infants exhibited elevated levels of selenium and iron.

We obtained higher levels of calcium in breastfed infants. In addition to being a vital component of bone, calcium is a messenger in cell-signaling pathways. Total calcium contents in breast milk rise significantly during the first five days of lactation, then gradually decrease over this course [12]. On the other hand, the concentration of ionized calcium in breast milk remains constant during the lactation process, indicating a homeostasis similar to that found in blood [13]. It has been reported that breast milk calcium concentrations are not impacted by maternal status [14] or vitamin D or calcium-containing diet interventions [15]. Therefore, it seems that despite the use of the elimination method in breastfed infants, we did not notice reduced calcium levels. It is noteworthy that breast milk typically contains about 200–250 mg/L of calcium. Our study was conducted on infants consuming an extensive hydrolyzed formula, which includes 610 mg/L of calcium.

In addition, the homeostasis of calcium can be kept by the hormone activities, including parathyroid hormone, 1, 25 dihydroxyvitamin D3, and calcitonin, which regulate its transportation in the intestine, kidneys, and bone. Therefore, it is assumed that not only the calcium intake from the diet can affect its level, but also these hormones can have a significant impact [16]. However, further investigations are needed to elucidate this issue.

We found that the mean serum level of selenium in both groups was in the normal range despite its significantly higher level in formula-fed infants. Selenium as a cofactor is necessary to preserve the glutathione peroxidase activity and it is a critical trace element in our body [17]. Colostrum has the largest quantities of selenium, which decreases as breastfeeding goes on. This pattern is similar to what is seen in milk proteins that contain selenium [18]. While breast milk typically contains about 1-2.5 micrograms/dL selenium. Our study was conducted on infants consuming an extensive hydrolyzed formula, which includes 3 micrograms/dL [19].

Several investigations have reported a considerable but weak correlation between selenium in blood plasma and breast milk [20, 21]. Breast milk selenium concentrations are highly influenced by dietary selenium intake, which is indicative of the selenium level in the soils used to farm food [22]. This variance explains why selenium concentrations have been found in a wide variety of geographical locations [23]. Therefore, it seems that we can consider the geographical regions we have performed our study as a probable cause of lower levels of selenium in breastfed infants despite it being placed in the normal range.

In our recent investigation, we observed that Iron (Fe) levels were notably lower in breastfed infants, though still within the normal range. Infant formulas are enriched with iron regarding the probability of iron deficiency and limited iron absorption in formula-fed compared to breastfed infants [24]. While breast milk typically contains about 0.3 mg/L, our study was conducted on infants consuming an extensive hydrolyzed formula, which includes 7.2 mg/liter of iron. Another key distinction between breast milk and formula lies in the elevated concentration of lactoferrin, a bioactive protein that binds to iron [24]. Therefore, it has a high bioavailability [25].

It is noteworthy that despite the low iron concentration in human milk, it is believed to be unrelated to maternal iron status and remains unaffected by maternal diet or iron supplementation [25]. Since we found that iron level was in the normal range in both groups and formula-fed infants had higher levels of iron, it can be inferred that breast milk composition is optimal for infants’ development. However, clinicians have to consider the side effects of increased iron on neurodevelopmental parameters, growth, and infection [24].

Notably a previous investigation assessing adolescents consuming highly iron-enriched formulas during infancy after 16 years showed that they had weak problem-solving, processing, and quantitative reasoning skills and worse visual-motor integration [26]. This evidence emphasizes the importance of monitoring iron levels before administering iron supplements in infants.

## Conclusions

The findings of this research revealed a significant difference in calcium, selenium, and iron levels between formula-fed and breastfed infants, even though all variables were within the normal range for both groups. Therefore, this study solves our concern that maternal dairy restrictions will disrupt the intake of enough calcium for this group of infants. In light of these results, conducting further studies with a larger sample size and extended follow-up periods becomes imperative. Such activities would contribute to more robust findings and shed light on a potentially more favorable feeding pattern for infants with CMA, promoting optimal growth and preventing malnutrition in children.

## Data Availability

The datasets generated during and/or analyzed during the current study are available from the corresponding author on reasonable request. All methods were carried out in accordance with relevant guidelines and regulations.

## References

[1] Iweala OI, Choudhary SK, Commins SP (2018). Food allergy. Curr Gastroenterol Rep.

[2] Rajani PS, Martin H, Groetch M, Järvinen KM (2020). Presentation and management of food allergy in breastfed infants and risks of maternal elimination diets. J Allergy Clin Immunology: Pract.

[3] Zhang S, Sicherer S, Berin MC, Agyemang A. Pathophysiology of non-IgE-mediated food allergy. ImmunoTargets and Therapy. 2021:431–46. 10.2147/ITT.S284821 PMC8721028 35004389

[4] Calvani M, Anania C, Cuomo B, D’Auria E, Decimo F, Indirli GC (2021). Non–IgE-or mixed IgE/non–IgE-mediated gastrointestinal food allergies in the first years of life: Old and new tools for diagnosis. Nutrients.

[5] Geiselhart S, Podzhilkova A, Hoffmann-Sommergruber K (2021). Cow’s milk processing—friend or foe in food allergy?. Foods.

[6] Plows JF, Berger PK, Jones RB, Alderete TL, Yonemitsu C, Najera JA (2021). Longitudinal changes in human milk oligosaccharides (HMOs) over the course of 24 months of lactation. J Nutr.

[7] Scherbaum V, Srour ML (2016). The role of breastfeeding in the prevention of childhood malnutrition. Hidden Hunger.

[8] Li R, Ware J, Chen A, Nelson JM, Kmet JM, Parks SE et al. Breastfeeding and post-perinatal infant deaths in the United States, a national prospective cohort analysis. Lancet Reg Health–Americas. 2022;5. 10.1016/j.lana.2021.100094 PMC9335131 35911656

[9] D’Auria E, Salvatore S, Acunzo M, Peroni D, Pendezza E, Di Profio E (2021). Hydrolysed formulas in the management of cow’s milk allergy: new insights, pitfalls and tips. Nutrients.

[10] Matthai J, Sathiasekharan M, Poddar U, Sibal A, Srivastava A, Waikar Y (2020). Guidelines on diagnosis and management of cow’s milk protein allergy. Indian Pediatr.

[11] Coppola S, Carucci L, Oglio F, Di Sarra C, Ozen G, Berni Canani R (2023). Nutritional strategies for the Prevention and Management of cow’s milk allergy in the Pediatric Age. Nutrients.

[12] Wei M, Deng Z, Liu B, Ye W, Fan Y, Liu R (2020). Investigation of amino acids and minerals in Chinese breast milk. J Sci Food Agric.

[13] Kovacs CS (2020). Physiology of calcium, phosphorus, and bone metabolism during pregnancy, lactation, and postweaning.

[14] Sánchez C, Fente C, Barreiro R, López-Racamonde O, Cepeda A, Regal P (2020). Association between breast milk mineral content and maternal adherence to healthy dietary patterns in Spain: a transversal study. Foods.

[15] Willemse JP, Meertens LJ, Scheepers HC, Achten NM, Eussen SJ, van Dongen MC (2020). Calcium intake from diet and supplement use during early pregnancy: the expect study I. Eur J Nutr.

[16] Yu E, Sharma S, Physiology. calcium. 2018. 29489276

[17] Islam MR, Akash S, Jony MH, Alam MN, Nowrin FT, Rahman MM et al. Exploring the potential function of trace elements in human health: a therapeutic perspective. Mol Cell Biochem. 2023:1–31. 10.1007/s11010-022-04638-3 36637616

[18] Riaz R, Ramay M, Tahir M, Rehman T, Ehsan M. A recent examination of the impact of selenium supplementation to lactating cattle. One Health Triad, Unique Scientific Publishers, Faisalabad, Pakistan. 2023;2:176 – 82.

[19] Kim SY, Yi DY (2020). Components of human breast milk: from macronutrient to microbiome and microRNA. Clin Experimental Pediatr.

[20] Dror DK, Allen LH (2018). Overview of nutrients in human milk. Adv Nutr.

[21] Plasma micronutrient concentrations (2015). Are altered by antiretroviral therapy and lipid-based nutrient supplements in lactating HIV-infected Malawian women. J Nutr.

[22] Daniels L, Haszard JJ, Gibson RS, Taylor RW, Fleming EA, Miller JC (2023). Selenium intakes and plasma selenium of New Zealand toddlers: secondary analysis of a randomised controlled trial. Br J Nutr.

[23] Genchi G, Lauria G, Catalano A, Sinicropi MS, Carocci A (2023). Biological activity of selenium and its impact on human health. Int J Mol Sci.

[24] Björmsjö M, Hernell O, Lönnerdal B, Berglund SK (2020). Reducing iron content in infant formula from 8 to 2 mg/l does not increase the risk of iron deficiency at 4 or 6 months of age: a randomized controlled trial. Nutrients.

[25] Friel J, Qasem W, Cai C (2018). Iron and the breastfed infant. Antioxidants.

[26] East PL, Reid B, Blanco E, Burrows R, Lozoff B, Gahagan S (2023). Iron supplementation given to nonanemic infants: neurocognitive functioning at 16 years. Nutr Neurosci.

